# Association of postoperative infection and oncological outcome after breast cancer surgery

**DOI:** 10.1093/bjsopen/zrab052

**Published:** 2021-07-08

**Authors:** L Adwall, E Pantiora, H Hultin, O Norlén

**Affiliations:** Department of Surgical Sciences, Uppsala University, Uppsala, Sweden; Department of Surgical Sciences, Uppsala University, Uppsala, Sweden; Department of Surgical Sciences, Uppsala University, Uppsala, Sweden; Department of Surgical Sciences, Uppsala University, Uppsala, Sweden

## Abstract

**Background:**

Surgical-site infection (SSI) is a well known complication after breast cancer surgery and has been reported to be associated with cancer recurrence. The aim of this study was to investigate the association between SSI and breast cancer recurrence, adjusting for several known confounders. The secondary aim was to assess a possible association between any postoperative infection and breast cancer recurrence.

**Method:**

This retrospective cohort study included all patients who underwent breast cancer surgery from January 2009 to December 2010 in the Uppsala region of Sweden. Data collected included patient, treatment and tumour characteristics, infection rates and outcome. Association between postoperative infection and oncological outcome was examined using Kaplan–Meier curves and Cox regression analysis.

**Results:**

Some 492 patients (439 with invasive breast cancer) with a median follow-up of 8.4 years were included. Mean(s.d.) age was 62(13) years. Sixty-two (14.1 per cent) of those with invasive breast cancer had an SSI and 43 (9.8 per cent) had another postoperative infection. Some 26 patients had local recurrence; 55 had systemic recurrence. Systemic recurrence was significantly increased after SSI with simple analysis (log rank test, *P* = 0.035) but this was not observed on adjusted analysis. However, tumour size and lymph node status remained significant predictors for breast cancer recurrence on multiple regression. Other postoperative infections were not associated with recurrence.

**Conclusion:**

Neither SSI nor other postoperative infections were associated with worse oncological outcome in this study. Rather, other factors that relate to both SSI and recurrence may be responsible for the association seen in previous studies.

## Teaser

Surgical site infection (SSI) is a well-known complication after breast cancer surgery and some previous studies suggest that SSI may increase the risk for breast cancer recurrence. In this population-based, retrospective cohort study we wanted to examine this possible association and also investigate if other postoperative infections were associated with breast cancer recurrence. The present study does, however, not support the hypothesis that postoperative SSI increases the rate of breast cancer recurrence.

## Introduction

In 2018 more than two million women were diagnosed with breast cancer and 627 000 died, making it the most common cancer in women worldwide^1^. The curative treatment for breast cancer is surgery, performed either as breast-conserving surgery (BCS) or mastectomy. The type of surgery chosen depends on, but is not limited to, tumour size, breast size, suitability for postoperative radiation and patient preference. Nowadays, most surgeons strive to achieve BCS, and there are only two absolute contraindications for this approach: failure to achieve negative margins without causing breast deformity and inflammatory breast cancer^2^. In conjunction with surgery of the breast, sentinel node surgery or lymph node clearance of the axilla is performed for staging. Adjuvant oncological treatment is essential to reduce recurrence and breast cancer-specific mortality^3–5^. Breast surgery-specific complications can compromise quality of life, increase costs and delay administration of adjuvant treatment. The most common complication after breast surgery is seroma. Haematoma, surgical-site infection (SSI) and chronic neuropathic postoperative pain are other well known complications^6^.

The postoperative SSI rate after breast cancer surgery varies between 0 and 19 per cent^7^. Factors that influence the rate of SSI are age, obesity, diabetes mellitus, smoking and recent chemotherapy. Hypertension, ASA score 3 or 4, a history of previous breast surgery, haematoma and lengthy or bilateral procedures have also been reported to increase the risk^6^. There are many reasons to reduce SSIs after breast cancer surgery. SSI can delay the start of adjuvant treatment, cause morbidity, increase costs and lead to failed reconstructions. Furthermore, some data suggest that SSI may increase the risk of breast cancer recurrence. Murthy and colleagues showed an increased risk of systemic recurrence in patients with wound complications than in those without^8^ and Beecher and co-workers demonstrated a six-fold higher risk for breast cancer recurrence (86 per cent had systemic recurrence) in patients with SSI following immediate breast reconstruction^9^. Another recently published study regarding postoperative wound complications and oncological outcome had findings in line with Beecher and co-workers^10^. However, although these studies do not provide a causal relationship between SSI and breast cancer recurrence, there is a theoretical link between the two. As early as 1863, Rudolf Virchow discovered white blood cells in malignant tissue and made the conclusion that there is a connection between inflammation and cancer^11^. The pro-tumour actions of inflammatory cells include releasing growth factors, promoting angiogenesis and lymph angiogenesis, stimulating DNA damage, remodelling the extracellular matrix to facilitate invasion, coating tumour cells to make receptors available for disseminating cells through lymphatics and capillaries and away from host defence mechanisms^12^. Therefore, a postoperative SSI with its inflammatory response could theoretically stimulate subclinical micrometastases and promote recurrence. This theory is supported with other malignancies where, for example, infection after colon cancer surgery increases the risk for recurrence^13^. Also, infectious complications after surgery for head and neck and gastric cancer correlate to worse outcome^14^^,^^15^.

Thus, although SSI may increase the risk for breast cancer recurrence, the studies may lack important data on confounding factors. Furthermore, any association of infections other than SSI and breast cancer recurrence has not been studied. In this study, the aim was to assess if the risk of systemic or locoregional breast cancer recurrence was increased after any postoperative infection, including SSI, while controlling for confounding variables such as co-morbidity, tumour and other patient characteristics.

## Methods

### Data collection

The electronic patient records for all three hospitals within the Uppsala region were scrutinized for all ICD codes for BCS and/or mastectomy (HAB00, HAB40, HAB99, HAC20, HAC22, HAC99). All patients with the above codes were included and a retrospective data set was assembled based on information in the patient charts. If the patient relocated, patient records were retrieved from the new hospital in charge of follow-up. Patients were followed up until death or October 2018. The article was written in accordance with the STROBE guidelines^16^.

### Patients

In this retrospective cohort study, all patients who underwent BCS or mastectomy to treat breast cancer between January 2009 and December 2010 were included. Both patients with contralateral breast cancer and those undergoing surgery because of local recurrence were included. Patients with distant metastasis at surgery or within 3 months from surgery and those having other cancer at pathological anatomical diagnosis were excluded.

For patients with bilateral tumours, the most aggressive tumour and most extensive surgery were included in the analysis. In cases of involved margins and subsequent reoperation, the tumour size was calculated from both surgeries. If a patient first underwent sentinel node biopsy and subsequent axillary clearance within 90 days, the total number of metastatic nodes was documented.

### Exposures, outcome and predictors

All patients underwent breast surgery, and the exposure was defined as SSI, any other postoperative infection or no infection within 90 days from surgery. SSI was defined as treatment with antibiotics and/or drainage due to erythema or purulent discharge with or without fever. Thus, according to the Clavien–Dindo classification, only SSIs grade II or higher were included. White blood cell count and C-reactive protein were not routinely measured to diagnose SSI. Other infections were registered when patients received antibiotic treatment without erythema or purulent discharge from the breast. Other infections included fever of unknown origin, urinary tract infection, other skin infection, pneumonia, tonsillitis, sepsis, sinusitis, otitis, diverticulitis and dental infection.

The primary outcome was systemic recurrence of breast cancer (including fossa supraclavicularis) and in this analysis patients operated on for *in situ* tumours were excluded. The secondary outcome included locoregional recurrence, defined as recurrence in the ipsilateral breast or axilla, breast cancer-specific survival and overall survival. Recurrence was defined as biopsy-proven recurrence or unequivocal radiology at any site noted in the patient records.

Predictors were age at surgery, body mass index (BMI), smoking status, diabetes, neoadjuvant chemotherapy, number of surgeries in the breast/axilla between 2009 and 2010, type of breast and axillary surgery, seroma aspiration, adjuvant chemotherapy, radiotherapy and hormone therapy, tumour size on pathology, tumour type, tumour grade, histological subtype and lymph node status. If not ductal or lobular tumour type, the tumour was classified as ‘other type’. To distinguish between luminal A and B subtypes, the Ki67 index and progesterone positivity was used: if low, or intermediate Ki-67 and positive for progesterone the tumour was classified as luminal A; if high, or intermediate Ki-67 and progesterone negative it was classified as luminal B. Antibiotic prophylaxis (flucloxacillin or clindamycin) was not given routinely but limited to those considered to have a high risk of SSI: neoadjuvant chemotherapy, reoperation, reconstruction or patients with specific risk factors. At the time of patient inclusion, the local clinical routine limited the use of neoadjuvant therapy to those with unresectable or borderline-resectable primary tumour.

### Statistical analysis

Descriptive data are presented as numbers with percentages and mean(s.d.). Univariable analysis of the exposure’s effect on distant and local breast cancer recurrence was performed by the Kaplan–Meier method and the log rank test. The association between exposure, predictors and recurrence was also analysed using simple Cox regression. Multiple Cox regression was performed to adjust the exposure for confounding predictors. Factors that proved significant on simple analysis were included in multivariable analysis. Results are presented as hazard ratios (HR) with 95 per cent confidence intervals. Overall survival, breast cancer-specific survival and distant recurrence-free survival were calculated with the Kaplan–Meier method.

A power calculation was made based on findings from previous studies^7–9^. The following parameters were considered: an SSI rate after breast cancer surgery of 10 per cent, and a hazard ratio of 2.5 for developing systemic recurrence in patients with SSI compared with no SSI. A sample size of 350 patients was calculated with a power 0.80 and type I error of 0.05.

All analyses were performed using SPSS^®^ version 25 (IBM, Armonk, New York, USA). *P* < 0.050 was considered statistically significant.

### Ethical considerations

The study was approved by the Regional Ethical Committee at Uppsala University (DNR 2018/312).

## Results

**Fig. 1:**
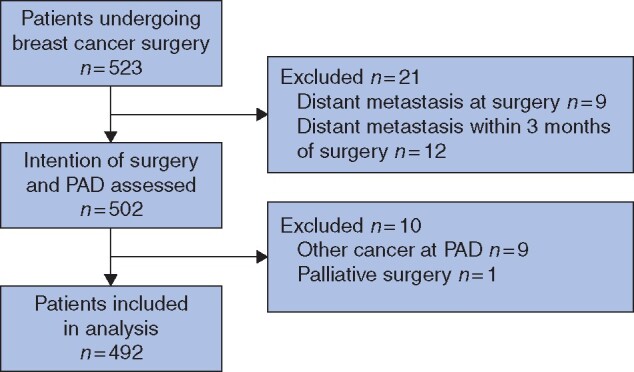
Flow chart PAD, pathological anatomical diagnosis

A total of 523 cases were included. Patients with distant metastasis at surgery or within 3 months of surgery, those revealed to have metastasis in the breast, those with sarcoma or malignant phylloides rather than primary breast cancer from the pathology result or those operated on without curative intent were excluded (*Fig. 1*). Thus, the study cohort comprised 492 patients. Of those, 10 patients had bilateral disease. Of the 482 patients with unilateral breast cancer, 30 patients were operated on due to local recurrence after previous ipsilateral breast cancer surgery. Fifty-five patients had a reoperation within 90 days either for non-radical breast surgery, additional axillary surgery for staging or postoperative haematoma.

Patient characteristics are shown in *Table 1,* treatment characteristics in *Table 2* and post-surgery pathology and infection rates in *Table 3.* Mean(s.d.) age was 62(13) years, ranging from 29 to 94 years. Mean(s.d.) BMI was 25.8(4.8), ranging from 15.4 to 48.8. Of the study cohort, 439 patients had invasive breast cancer and 53 *in situ* tumours. Antibiotic prophylaxis was given to 52 (10.6 per cent) patients. Four patients underwent immediate reconstruction. Median (range) follow-up was 8.4 (0.2–10.1) years.

**Table 1:** Patient characteristics

Patient variables	Total cohort (*n* = 492)
**Age at surgery (years)**	
≤40	25 (5.1)
41–55	148 (30.1)
56–70	194 (39.4)
>70	125 (25.4)
**BMI (kg/m^2^)**	
<18.5	13 (2.6)
18.5–24.9	215 (43.7)
25–29.9	157 (31.9)
>30	86 (17.5)
Missing data	21 (4.3)
**Smoker**	
Yes	81 (16.5)
Previous	123 (25.0)
No	255 (51.8)
Missing data	33 (6.7)
**Diabetic**	
Yes	22 (4.5)
No	470 (95.5)

Values in parentheses are percentages.

**Table 2:** Treatment characteristics

Treatment variables	**Total cohort** **(*n* = 492)**
**Neoadjuvant chemotherapy**	
Yes	9 (1.8)
No	483 (98.2)
No. of surgeries (breast/axilla)*	
1	421 (85.6)
>1	71 (14.4)
**Baseline breast surgery**	
Breast-conserving surgery	298 (60.6)^†^
Mastectomy	194 (39.4)
**Baseline axillary surgery**	
Sentinel node biopsy	278 (56.5)^‡^
Axillary clearance	154 (31.3)
No surgery	60 (12.2)^§^
**Seroma aspiration**	
Yes	63 (12.8)
No	429 (87.2)
**Adjuvant chemotherapy**	
Yes	170 (34.6)
No	322 (65.4)
**Adjuvant radiotherapy**	
Yes	309 (62.8)
No	183 (37.2)^¶^
**Adjuvant hormone therapy**	
Yes	337 (68.5)
No	155 (31.5)

Values in parentheses are percentages.

*During years 2009–2010. ^†^Additional mastectomy within 90 days (*n* = 19).

‡ Subsequent axillary clearance within 90 days (*n* = 3).

§ 37 patients had no axillary surgery (ductal carcinoma *in situ* (*n* = 17); age/co-morbidity (*n* = 19); own choice (*n* = 1)), 11 had previous axillary clearance but not at baseline, and 12 had subsequent axillary surgery within 30 days.

¶ Previous radiotherapy (n = 17).

**Table 3:** Pathology and infection rates after surgery

Tumour and infection variables	**Total cohort** **(*n* = 492)**
**Tumour size – invasive**	
T1	230 (52.4)
T2	177 (40.3)
T3	30 (6.8)
Missing data	2 (0.5)
**Tumour size *in situ* (mm)**	
≤20	26 (49.1)
21–50	21 (39.6)
>50	6 (11.3)
**Tumour type**	
Ductal	360 (73.2)
Lobular	54 (11.0)
Mixed	4 (0.8)
Other invasive types	20 (4.1)
DCIS	51 (10.4)
LCIS	2 (0.4)
Missing data	1 (0.2)
**Tumour grade**	
1	77 (17.5)
2	231 (52.6)
3	125 (28.5)
Missing data	6 (1.4)
**DCIS grade**	
1	4 (7.5)
2	18 (34.0)
3	25 (47.2)
Missing data	6 (11.3)
**Histological subtype**	
Luminal A	220 (44.7)
Luminal B	116 (23.6)
HER 2+ ER+	26 (5.3)
HER 2+ ER-	21 (4.3)
Triple negative	56 (11.4)
*In situ*	53 (10.8)
Lymph node status*	
N0	271 (61.7)
N1	92 (21.0)
N2	45 (10.3)
Missing data	31 (7.1)
**SSI**	
Yes	70 (14.2)
No	422 (85.8)
**Other infection**	
Yes	49 (10.0)
No	443 (90.0)
**SSI and/or other infection**	
Yes	113 (23.0)
No	379 (77.0)

Values are number (per cent).

*Only invasive breast cancer (439 patients). DCIS, ductal carcinoma *in situ*; LCIS, lobular carcinoma *in situ*; HER2, human epidermal growth factor receptor 2; ER, oestrogen receptor; SSI, surgical-site infection

Seventy (14.2 per cent) of all patients and 62 (14.1 per cent) of those with invasive breast cancer had an SSI. Forty-nine (10.0 per cent) of all patients and 43 (9.8 per cent) of those with invasive cancer had an infection other than SSI. Of the patients with other infection, 17 had fever of unknown origin or urinary tract infection, four had skin infection at another location, two had pneumonia, tonsillitis, sepsis or sinusitis and one had otitis, diverticulitis or dental infection.

Some 26 patients had local recurrence and 55 had systemic recurrence. The median (range) time to locoregional recurrence was 3.0 (0.7–10.1) years and to systemic recurrence 2.6 (0.4–10.1) years. Seven of the patients with systemic recurrence had also had or developed a contralateral breast cancer after surgery. The mean(s.d.) 5-year overall survival, breast cancer-specific survival and distant recurrence-free survival rates were 86.5(2.9), 94.6(2.0) and 90.6(2.9) per cent respectively.

**Fig. 2:**
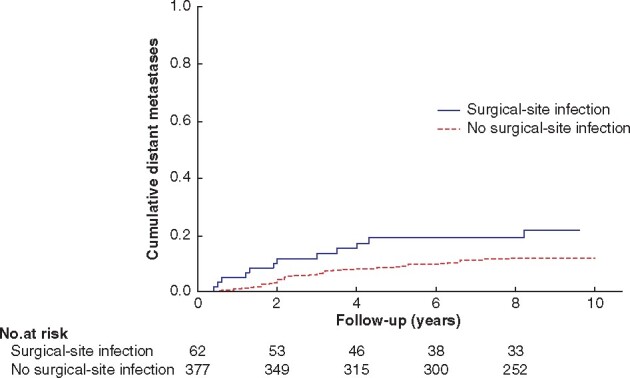
Kaplan–Meier analysis of distant recurrence in patients with and without surgical-site infection *P* = 0.035 (log rank test)

On unadjusted analysis, the risk of systemic recurrence was significantly increased after SSI (log rank test *P* = 0.035, *Fig. 2*) but SSI was not associated with the local recurrence rate (*P* = 0.310).

Other predictors significantly associated with systemic recurrence on univariable analysis were: age at surgery, receiving neoadjuvant chemotherapy, breast and axillary surgery, seroma aspiration, tumour size, tumour grade, histological subtype and lymph node status (*Table 4*).

**Table 4:** Unadjusted and adjusted Cox regression analysis of factors associated with systemic recurrence with a median of 8.4 years’ follow-up, based on 439 patients with invasive breast cancer

		Unadjusted results		Adjusted results	
Factors	No. of patients	Hazard ratio	*P*	Hazard ratio	*P*
Age at surgery	439	1.02 (1.00, 1.05)	**0.038**	1.02 (0.99, 1.04)	0.201
BMI (kg/m^2^)					
18.5–24.9	189	1.00 (reference)			
<18.5	12	0.00 (0.00, 0.00)	0.974		
25–29.9	143	1.09 (0.60, 2.01)	0.772		
>30	80	1.15 (0.56, 2.36)	0.699		
Smoker					
No	228	1.00 (reference)			
Yes	74	0.45 (0.16, 1.28)	0.134		
Previous	111	1.43 (0.80, 2.56)	0.222		
Diabetic					
No	420	1.00 (reference)			
Yes	19	1.00 (0.24, 4.10)	0.998		
Neoadjuvant chemotherapy					
No	430	1.00 (reference)		1.00 (reference)	
Yes	9	3.25 (1.01, 10.42)	**0.047**	1.53 (0.43, 5.47)	0.512
No. of surgeries (breast/axilla)*					
1	381	1.00 (reference)			
>1	58	0.82 (0.35, 1.91)	0.643		
Breast surgery					
Breast-conserving surgery	264	1.00 (reference)		1.00 (reference)	
Mastectomy	175	3.70 (2.09, 6.54)	**0.000**	1.42 (0.68, 2.98)	0.355
Axillary surgery					
Sentinel node biopsy	247	1.00 (reference)			
Axillary clearance	150	2.90 (1.63, 5.15)	**0.000**		
No surgery	42	1.71 (0.58, 5.03)	0.331		
Seroma aspiration					
No	381	1.00 (reference)		1.00 (reference)	
Yes	58	2.18 (1.15, 4.15)	**0.018**	0.84 (0.35, 2.03)	0.702
Adjuvant chemotherapy					
No	269	1.00 (reference)			
Yes	170	1.68 (0.98, 2.87)	0.061		
Adjuvant radiotherapy					
No	132	1.00 (reference)			
Yes	294	0.89 (0.48, 1.65)	0.710		
Previous radiotherapy	13	2.61 (0.86, 7.92)	0.091		
Adjuvant hormone therapy					
No	103	1.00 (reference)			
Yes	336	0.61 (0.34, 1.09)	0.095		
Tumour size					
T1	230	1.00 (reference)		1.00 (reference)	
T2	177	5.58 (2.67, 11,67)	**0.000**	2.78 (1.23, 6.24)	**0.014**
T3	30	13.00 (5.27, 32.06)	**0.000**	5.36 (1.89, 15.21)	**0.002**
Tumour type					
Ductal	360	1.00 (reference)			
Lobular	54	1.26 (0.59, 2.68)	0.547		
Other invasive types	25	0.32 (0.04, 2.34)	0.263		
Tumour grade					
1	77	1.00 (reference)		1.00 (reference)	
2	231	10.02 (1.36, 73.64)	**0.024**	4.10 (0.54, 31.17)	0.172
3	125	15.73 (2.12, 116.46)	**0.007**	3.90 (0.46, 32.80)	0.210
Histological subtype					
Luminal A	220	1.00 (reference)		1.00 (reference)	
Luminal B	116	2.01 (1.03, 3.94)	**0.041**	1.42 (0.68, 2.96)	0.348
HER 2+ ER+	26	2.06 (0.69, 6.11)	0.195	1.02 (0.27, 3.87)	0.974
HER 2+ ER-	21	3.38 (1.25, 9.17)	**0.017**	1.85 (0.50, 6.81)	0.358
Triple negative	56	2.54 (1.17, 5.56)	**0.019**	2.68 (0.98, 7.31)	0.054
Lymph node status					
N0	271	1.00 (reference)		1.00 (reference)	
N1	92	2.87 (1.47, 5.64)	**0.002**	2.21 (1.08, 4.52)	**0.031**
N2	45	8.04 (4.18, 15.47)	**0.000**	3.89 (1.77, 8.55)	**0.001**
SSI					
No	377	1.00 (reference)		1.00 (reference)	
Yes	62	1.97 (1.04, 3.76)	**0.038**	0.99 (0.41, 2.34)	0.972
Other infection					
No	390	1.00 (reference)			
Yes	49	1.57 (0.73, 3.30)	0.249		
SSI and/or other infection					
No	334	1.00 (reference)			
Yes	105	1.79 (1.02, 3.17)	**0.044**		

Values in parentheses are 95 per cent confidence intervals.

*During years 2009–2010. HER2, human epidermal growth factor receptor 2; ER, oestrogen receptor; SSI, surgical-site infection. Bold numbers are significant *P* values.

SSI did not predict the rate of systemic recurrence on multiple Cox regression analysis. However, tumour size and lymph node status remained significant predictors on multiple regression. Axillary metastasis, but not axillary surgery, was included in the multivariable analysis due to multicolinearity, since all patients with axillary metastasis also had undergone axillary clearance. A sensitivity analysis excluding patients that received antibiotic prophylaxis was performed, however this multivariable analysis including the same factors as the multivariable analysis in *Table 4* did not show any significant differences in outcome.

Infection other than SSI was not associated with the rate of systemic (hazard ratio 1.57, *P* = 0.249) or local (hazard ratio 2.49, *P* = 0.068) recurrence on univariable analysis and further multivariable testing was thus not performed.

## Discussion

The most important finding in this study was that postoperative SSI was associated with systemic recurrence on crude analysis, but not in the adjusted multivariable analysis, suggesting that confounding influences the risk for recurrence. Moreover, SSI was not correlated with local recurrence, and infection other than SSI did not affect local or distant recurrence.

In contrast to this study, Murthy and colleagues demonstrated a significantly increased risk of systemic recurrence in patients with a postoperative wound complication compared with those without^8^. However, several differences in definition of exposure, predictors and outcome exist that may explain these conflicting results. For example, Murthy and colleagues defined a wound complication as ‘any wound breakdown that occurred before completion of adjuvant chemotherapy and radiotherapy and that needed surgical debridement, dressing, or packing or any persistent discharge from the wound’^8^. In contrast to the current study, erythema alone of the wound was not included and length of SSI follow-up was not clearly defined. Thus, there was a wider definition of SSI in the present study, however other wound complications were not included. Murthy and colleagues also used the Nottingham Prognostic Index (NPI; good, intermediate and poor), calculated from histological grade 1, 2 or 3 + nodal status (no positive nodes = 1, 1–3 nodes = 2 and more than 3 nodes positive = 3) + 0.2 × size of tumour in centimetres, while in the present study those predictors were calculated one by one. Another difference is that in the study by Murthy and colleagues, all patients went through axillary clearance, whereas in the present study most patients underwent sentinel lymph node biopsy only, which may have influenced the results. Beecher and co-workers describe a majorly increased risk for recurrence if the patient has an SSI after breast cancer surgery with immediate reconstruction (hazard ratio 6.15 (95 per cent c.i. 3.33 to 11.33))^9^. The local clinical routine at the time of the study was to perform late reconstruction as a second procedure and not concomitant with the breast cancer surgery. In the present study, only four immediate reconstructions were made, so in this aspect these two cohorts are not comparable. However, Beecher and co-workers also used NPI groups and not tumour grade, lymph nodal status and tumour size separately, which also may explain the diverging results from the present study. Complications considered for their analysis were wound infection (cellulitis, purulent discharge or abscess), haematoma formation, flap dehiscence or skin necrosis that developed within 30 days after surgery. Thus, the definition of complication also differs between the studies. Two other studies did not demonstrate any association between postoperative complications and recurrence in breast cancer patients who received mastectomy with immediate reconstruction^17^^,^^18^.

Axillary clearance seems to increase the risk for postoperative SSI, in this cohort three times higher than for breast surgery without axillary surgery. In the present study, both axillary surgery and advanced axillary nodal status increased the risk of systemic recurrence. Hence, this finding may explain that SSI demonstrated an increased risk for recurrence in the univariable analysis but not in the multivariable analysis. In the present study, tumour size and lymph node status significantly increased the risk for systemic recurrence on multivariable analysis. This conforms with a recently published large registry study from Germany^19^. Common to both of these studies, Murthy and co-workers and Beecher and colleagues describe that tumour size and lymph node status (measured as NPI index) significantly affect the risk for systemic recurrence^8^^,^^9^. Regarding triple-negative subtype (borderline significant in the present study), Beecher and colleagues analysed it, but since it was not statistically significant on crude analysis it was not included in multiple Cox regression^9^. Also, data from the population-based Saarland Cancer Registry, including 9359 female patients with primary invasive breast cancer between 1999 and 2009, demonstrate that the risk for recurrence (both locoregional and systemic) is particularly increased if tumours are locally or regionally advanced (T3/4 N+), of high grade or classified as subtype HER 2-positive without hormone receptor expression or triple negative. Patients aged less than 70 years also have higher risk for recurrence^19^.

Another theory is that surgery or other interventions suppress cell-mediated immunity and increase the risk for recurrence^20^. A study from 2011 describes a significantly increased risk of recurrence after mastectomy followed by delayed reconstruction with autologous tissue compared with mastectomy only^21^. However, two other studies were published demonstrating that delayed deep inferior epigastric perforator flap reconstruction after breast cancer surgery did not increase the risk of recurrence compared with mastectomy alone^22^^,^^23^. In the present study there was no increased risk for recurrence among those who had more than one surgery compared with those who had only one operation. In a cohort study using the Danish National Patient Register with more than 30 000 patients, there was no association between reoperation for bleeding and breast cancer recurrence^24^ also questioning the association of bleeding, inflammation and cancer recurrence.

The strength of this study is the relatively long follow-up of all cases^8^^,^^9^^,^^17^. One limitation relates to the true incidence of SSI in the absence of wound cultures in many cases. However, there is no reason to believe this limitation is causing a systemic bias. The present study does not support the hypothesis that postoperative SSI after breast cancer surgery increases the rate of locoregional or systemic breast cancer recurrence. Rather, above-mentioned factors that relate to both SSI and recurrence may be responsible for the association seen in previous studies. To investigate the conflicting results of this and previous studies further, the best option would be a prospective multicentre study where data on unequivocal infections (those with positive wound culture) and long follow-up are available. Furthermore, future studies should study any interventions to lessen the risk of postoperative infection and, thus, systemic recurrence if an association is found.
